# The Limitation of Endoscopic Surgery Using the Full Endoscopic Discectomy System for the Treatment of Destructive Stage Pyogenic Spondylodiscitis: A Case Series

**DOI:** 10.1155/2021/5582849

**Published:** 2021-11-25

**Authors:** Tomoyuki Setoue, Jun-Ichiro Nakamura, Jun Hara

**Affiliations:** Saiwai Tsurumi Hospital, 1-21 Toyooka, Tsurumi, Yokohama 230-0062, Japan

## Abstract

**Introduction:**

Conservative therapy, including appropriate antibiotics and bracing, is usually adequate for most patients with pyogenic spondylodiscitis. If conservative treatment fails, surgical intervention is needed. However, major spinal surgery comprising anterior debridement and accompanying bone grafting with or without additional instrumentation is often related to undesired postoperative complications. In recent years, with minimally invasive surgery, the diagnostic and therapeutic value of endoscopic lavage and drainage has been proven. This study reports a case series of patients who required open revision surgery after treatment with endoscopic surgery using the full endoscopic discectomy system (FED), indicating the surgical limitations of endoscopic surgery for pyogenic spondylodiscitis.

**Methods:**

We retrospectively investigated the medical records of 4 patients who underwent open debridement and anterior reconstruction with posterior instrumentation following endoscopic surgery for their advanced lumbar infectious spondylitis. They had been receiving conservative treatment with antibiotics for 12–15 days. They also had various comorbidities, including kidney disease, heart failure, and diabetes. Numerical rating scale pain response, perioperative imaging studies, and C-reactive protein (CRP) levels were determined, and causative bacteria were identified. Primarily, the bone destruction stage was classified using computed tomography with reference to Griffiths' scheme.

**Results:**

All patients had severe back pain before surgery with no relief of the pain after FED. Increased pain, including radicular pain after FED, was noted in one case. Causative pathogens from biopsy specimens were identified in 3 (75%) of the 4 cases. In preoperative radiological evaluation, all cases were classified as destructive stage in Griffiths' scheme. The CRP levels of all the patients decreased slightly after endoscopic surgery. Relapse of spinal infection after revision surgery was not noted in any patient during the follow-up period.

**Conclusion:**

The surgical treatment of destructive-stage spondylitis with FED alone can increase low back pain due to aggressive debridement.

## 1. Introduction

Recently, the percentage of the elderly in the overall population and the number of immunocompromised hosts have been increasing, thereby resulting in an upward trend in the incidence of pyogenic spondylitis. Conservative treatment using antibiotics and bracing is usually adequate for most patients. Poor response to conservative treatment, severe persistent low back pain with deformity and instability, and progressing neurological deficits are indications for surgical treatment. However, major spinal surgery comprising anterior debridement with bone grafting is often related to postoperative complications [1]. In recent times, several minimally invasive methods, such as computed tomography-guided debridement and drainage, percutaneous discectomy, and procedures involving percutaneous pedicle screws have been used to treat infectious spondylitis [2–6].

The clinical outcomes of posterolateral spinal endoscopic surgery for pyogenic spondylodiscitis have already been reported in some recent studies, thus demonstrating satisfactory outcomes [7–10].

The purpose of this study is to report a case series of patients with pyogenic spondylodiscitis that was unsuccessfully treated with endoscopic surgery using the full endoscopic discectomy system (FED).

## 2. Materials and Methods

### 2.1. Demographic Data of the Patients

We retrospectively examined 4 patients (2 males and 2 females; mean age, 70.6 years) with pyogenic lumbar spondylodiscitis who underwent debridement and anterior fusion surgery following FED. The mean follow-up period was 19.5 months (6–26 months). The organisms had been identified in all cases by blood cultures, and patients had been receiving conservative treatment with compatible antibiotics for 12–15 days at the Department of Internal Medicine. Despite previous conservative treatment, their spinal infection had not been controlled and they were referred to our department. All the patients presented with severe low back pain, requiring narcotic pain control and bed rest. They also had various comorbidities, including kidney disease, heart failure, and diabetes.

The affected level ranged from L2 to L5 in all the cases. Infectious spondylitis was diagnosed based on clinical examination, including elevated erythrocyte sedimentation rate and C-reactive protein (CRP) values, which are inflammation markers, and roentgenographic and magnetic resonance imaging findings. The perioperative bone destruction stage was classified using computed tomography with reference to Griffiths' scheme as early stage (narrowing of the disc space), destructive stage (bone destruction, collapse of softened vertebrae, and bone proliferation), and osteosclerotic stage (new bone formation and osteosclerotic changes) [11]. Perioperative clinical outcome was evaluated using a numerical rating scale (NRS). During the endoscopic procedure, infected necrotic tissue was collected to detect the causative organisms. CRP values were recorded preoperatively, postoperatively on day 5, and postoperatively on day 10, and computed tomography during admission.

### 2.2. Operative Procedure

The FED procedures were performed as previously described [10, 12, 13]. All procedures were carried out under total anesthesia, monitoring motor-evoked potentials during surgery to prevent nerve damage. The patients were placed prone on a radiolucent surgical bed. After creating 10 mm skin incision at a lateral point about 10 cm from the midline, a spinal needle was inserted directly into the affected intervertebra under fluoroscopic guidance. A single portal was placed except for one case with marked abscess retention. Percutaneous debridement of infected disc material and vertebral bodies was conducted using several types of rongeurs through the portal. Cultures of the organisms were taken from these resected materials to identify the organisms and effective antibiotics. Then, irrigation with more than 2000 ml saline was performed. Finally, a drainage catheter was inserted into the debrided disc space.

## 3. Results

**Figure 1:**
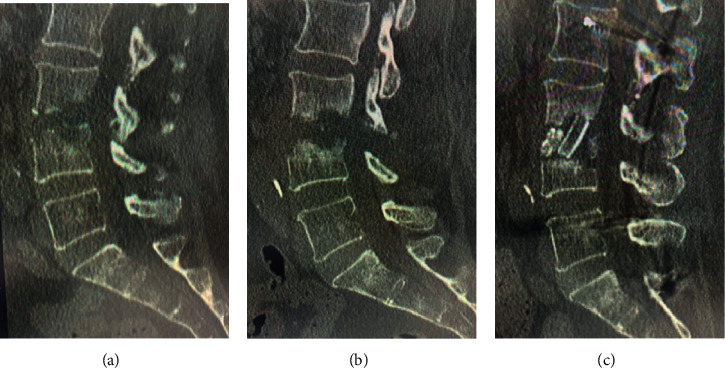
A 70-year-old female was diagnosed with L3/L4 infectious spondylitis and underwent endoscopic debridement, although the pain was increased postoperatively. She underwent anterior reconstruction surgery with iliac autograft. (a) The preoperative sagittal CT scan view showing severe osteolytic changes at the L3/L4 level. (b) The post-FED sagittal CT scan view showing an exacerbated defect of the vertebra. (c) The post-open surgery sagittal CT scan view; the iliac strut bone was grafted.

**Figure 2:**
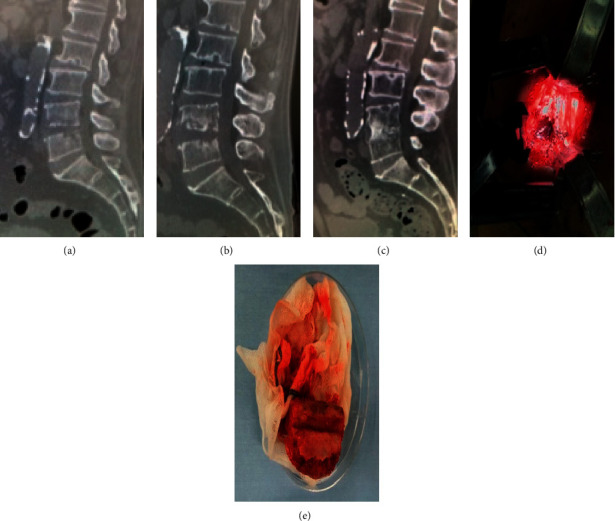
A 75-year-old male with L4/L5 pyogenic spondylitis underwent endoscopic debridement. Because low back pain did not improve postoperatively, anterior reconstruction surgery with an iliac autograft was performed. (a) Preoperative sagittal CT scans revealed destructive endplate at the L4/L5 level. (b) Post-endoscopic surgery sagittal CT scans showing further bone destruction. (c) Post-open surgery sagittal CT scan view showing the bony fusion without severe kyphotic deformity.(d, e) Intraoperative view and iliac autograft; minimally invasive surgery was performed via the direct lateral retroperitoneal approach, and the large defect due to debridement was reconstructed with the strut bone collected from the ilium.

**Figure 3:**
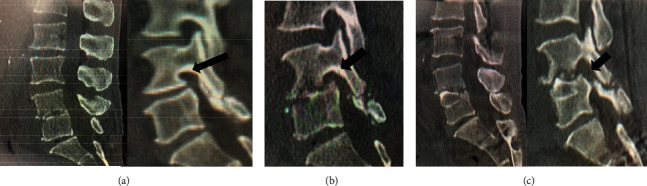
A 77-year-old male had severe back pain and could not sit for a long time due to pyogenic spondylodiscitis at the L4/L5 level. He underwent surgery with FED; however, not only did the postoperative pain scale scores remain unchanged, but also left thigh pain and quadriceps muscle weakness also appeared. He underwent anterior spinal fusion with an iliac strut autograft 13 days after FED. After the revision surgery, low back pain and neurological disorder improved. (a) Preoperative sagittal CT scan images showing destructive changes, including narrowing of disc height and destruction of the endplate at the L4/L5 level, although the foraminal sagittal view did not show foraminal stenosis (arrow). (b) Foraminal sagittal CT scan view after FED showing the progress of vertebral destruction and stenosis of the foramen (arrow). (c) Sagittal CT scan images showing regained foraminal space (arrow) after open surgery with anterior strut autograft.

**Figure 4:**
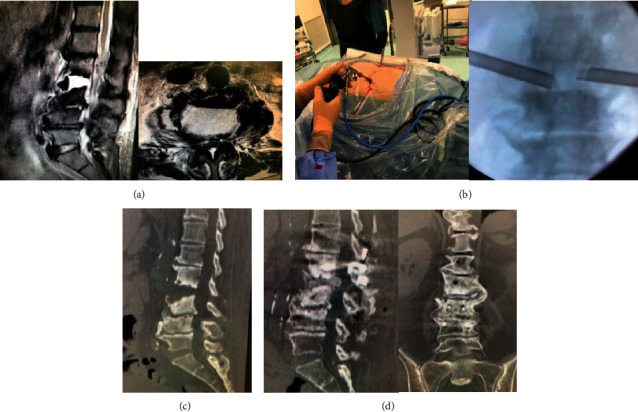
A 71-year-old female was referred because she had severe low back pain after treatment at the Department of Internal Medicine due to urinary tract infection. After spinal endoscopic surgery, low back pain persisted and the patient found difficulty in getting out of the bed; therefore, anterior-posterior lumbar fusion was performed. After the operation, the low back pain improved. (a) Sagittal and axial T2-weighted MRI; an abscess that had formed an air fluid level is identified at the L2/L3 level. (b) Intraoperative view and fluoroscopic image; two portals are inserted on both sides of the intervertebral space, and debridement and irrigation were performed endoscopically. (c) Post-FED sagittal CT scan image showing progress of bone destruction. (d) Post-open surgery sagittal and coronal CT scan view; bone fusion at L2/L3 level was observed 3 months after the minimally invasive open surgery.

All the patients had severe back pain (mean NRS score = 9.7) before surgery; increased pain, including the onset of radicular pain after FED, was noted in one case. In the other three cases, the NRS scores did not change (Table 1). The causative pathogens in biopsy specimens were identified in three (75%) of the four cases (Table 2); the isolated pathogens were *Escherichia coli*, Group B *Streptococcus*, and methicillin-susceptible *Staphylococcus aureus*. Preoperative radiological evaluation revealed that all the cases were classified as destructive stage in Griffiths' scheme and postoperative computed tomography showed further bone destruction (Figures 1−4). The CRP levels of all patients decreased slightly after endoscopic surgery (Table 3). Not all cases were able to walk due to pain after FED; therefore, they were indicated for anterior reconstruction surgery. The average duration from FED to anterior fusion was 18.5 (range, 10–24) days. No patient reported relapse of spinal infection after open revision surgery during the follow-up period.

## 4. Discussion

FED was first employed for the treatment of uncomplicated herniated discs in the early 1980s. The clinical outcomes of this procedure are comparable with those of conventional open surgery [12, 14]. The minimal invasiveness and simplicity of the technique led surgeons to use FED for spinal debridement and drainage and as a modality for treating infectious spondylodiscitis. In addition to the minimal invasiveness, bacteriologic specimens could be directly obtained from the infected lesion to identify the organisms and administer effective antibiotics. In our study, the organisms in the collected tissue during FED were detected in 3 cases (75%) even though compatible antibiotics had been started in all cases before surgery.

The diagnostic and therapeutic value of percutaneous endoscopic lavage and drainage has been proved [8–10], indicating the effectiveness of posterolateral spinal endoscopic surgery for pyogenic spondylodiscitis. However, most of these studies have focused on early-stage infection. Ito reported that all patients (*n* = 15) with pyogenic spondylodiscitis experienced immediate back pain reduction after surgery and that the infections were successfully treated with subsequent parenteral antibiotic treatment [10].In our study, the same posterolateral endoscopic technique was used to treat four patients with pyogenic spondylodiscitis. Given that not all patients experienced alleviation of back pain after surgery and underwent open surgery, including anterior reconstruction with iliac strut bone graft and posterior instrumentation (Figures 1−4), there are two possible causes for these adverse consequences. Firstly, all patients were compromised hosts with comorbidities such as diabetes. Secondly, it is possible that vertebral destruction has progressed because all patients had been receiving conservative therapy for some period. In particular, two cases showed severe intervertebral defects preoperatively (Figures 1 and 4). Furthermore, aggressive debridement with FED increased instability and induced exacerbated pain. In the worst cases, neurological disorders due to foraminal stenosis would appear, such as that noted in our study (Figure 3).

In addition to the postoperative progress of vertebral destruction, preoperative destructive changes at vertebral levels reportedly illustrated local kyphosis progression during follow-up due to aggressive debridement after FED [10, 13]. Thus, it is necessary to quantify and evaluate the degree of preoperative bone destruction and determine a clear indication of FED. When more extensive bone destruction is identified, open debridement and bone grafting can provide adequate stability, relieve symptoms, and prevent kyphosis. In recent times, the minimally invasive direct lateral retroperitoneal approach for the surgical treatment of lumbar discitis and osteomyelitis, which allows for thorough debridement and spinal reconstruction, has also been reported [15, 16].Therefore, factors such as progress of vertebral destruction may be considered as exclusion criteria for endoscopic procedures, indicating that it is better to perform open surgery in a minimally invasive manner for the primary treatment in such cases.

This study has several limitations. First, we examined only four cases; therefore, the results cannot be generalized. Second, the retrospective nature of this study lacks a random assignment of subjects and does not allow the enrolled patients to be subjected to different treatment methods for subsequent comparison of clinical outcomes. Finally, the absence of a control group made it difficult to validate the negative treatment effect of FED in destructive-stage spondylitis. In the future, we need further verification of the clinical results depending on the degree of bone destruction with a large sample size. In addition, guidelines for the surgical treatment of pyogenic spondylodiscitis should be established.

## 5. Conclusion

In destructive-stage spondylitis, surgical treatment with FED alone can promote bone destruction due to aggressive debridement, which can exacerbate postoperative low back pain. Further investigations with larger sample sizes to select the appropriate treatment depending on the degree of bone destruction are needed.

## Figures and Tables

**Table 1:** Perioperative pain scale (NRS), ADL, and neurological complications.

	Pre-NRS	Post-NRS	Pre-ADL	Post-ADL	Post-neurological disorder
(1)	10	10	Bed	Bed	—
(2)	10	10	Bed	Bed	—
(3)	10	10	Bed	W.C	Quad MMT3
(4)	8	10	Bed	W.C	—

Pre = before FED, post = after FED, ADL = activity of daily living, bed = bed rest, W.C = wheelchair, quad = quadriceps, and MMT = manual muscle test.

**Table 2:** Summary of data in four patients.

	Patient	Level	Organism	Comorbidity	Onset to FED (day)	FED to OS (day)	F/U (month)
(1)	75 m	L4/L5	*E. coli*	DM, CKD	15	14	22
(2)	71 f	L2/L3	GBS	CHF	14	15	26
(3)	77 m	L4/L5	MSSA	DM	13	14	6
(4)	70 f	L3/L4	—	DM	12	15	24

*E. coli* = *Escherichia coli*, GBS = group B *Streptococcus*, MSSA = methicillin-susceptible *Staphylococcus aureus*, DM = diabetes mellitus, CHF = chronic heart failure, CKD = chronic kidney disease, FED = full endoscopic discectomy, OS = open surgery, and F/U = follow-up.

**Table 3:** Perioperative change of CRP (mg/dL).

	Pre-FED	Post-FED day 5	Post-FED day 10
(1)	8	3	2.2
(2)	4	3	1.5
(3)	12.3	11.2	8.3
(4)	4.5	2.3	2.2

FED = full endoscopic discectomy and CRP = C-reactive protein.

## Data Availability

Derived data supporting the findings of this study are available from the corresponding author on request.
